# Naked-Eye Detection of LAMP-Produced Nucleic Acids in Saliva Using Chitosan-Capped AuNPs in a Single-Tube Assay

**DOI:** 10.1021/acs.analchem.3c03878

**Published:** 2023-12-08

**Authors:** Stylianos Grammatikos, Ioannis Svoliantopoulos, Electra Gizeli

**Affiliations:** † Institute of Molecular Biology and Biotechnology, Foundation for Research and Technology-Hellas, 100 N. Plastira Str., 70013 Heraklion, Greece; ‡ Department of Biology, University of Crete, Voutes, 70013 Heraklion, Greece; § Department of Chemistry, University of Crete, Voutes, 70013 Heraklion, Greece

## Abstract

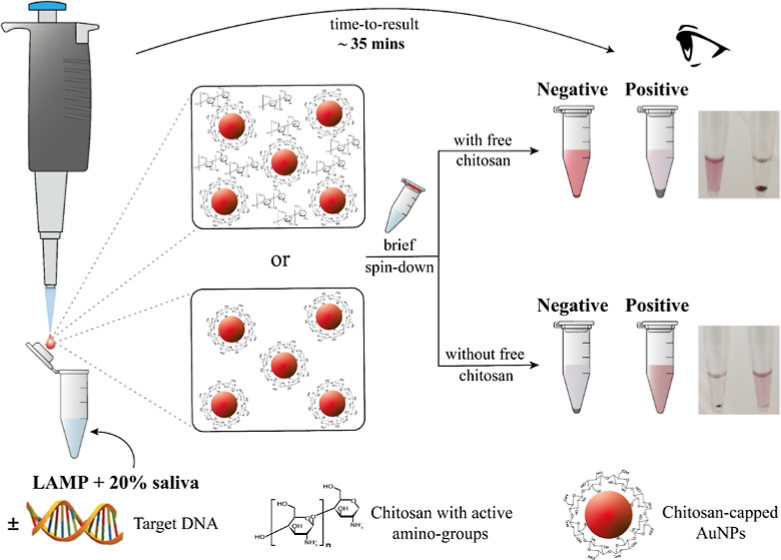

Loop-mediated isothermal amplification (LAMP) is a low-technology molecular assay that is highly adaptable to point-of-care (POC) applications. However, achieving sensitive naked-eye detection of the amplified target in a crude sample is challenging. Herein, we report a simple yet highly efficient and sensitive methodology for the colorimetric visualization of a single target copy in saliva using chitosan-capped gold nanoparticles (Chit-AuNPs) synthesized via a green chemistry approach. The presence or absence of free Chit in the Chit-AuNPs solution was shown to affect LAMP colorimetric detection oppositely: the observed stabilization in the negative samples and aggregation in the positive samples in the presence of free Chit were reversed in the case of neat Chit-AuNPs. The mechanism of the two assays was investigated and attributed to electrostatic and depletion effects exerted between the Chit-AuNPs, free Chit, and the solution components. The developed contamination-free, one-tube assay successfully amplified and detected down to 1–5 cfu of *Salmonella* and 10 copies of SARS-CoV-2 per reaction (25 μL) used, respectively, as model DNA and RNA targets in the presence of 20% saliva, making the method suitable for POC applications. Compared to the commonly used pH-sensitive dyes, Chit-AuNPs are shown to have an enhanced sensitivity toward naked-eye colorimetric observation owing to the direct detection of DNA amplicons. Thus, this is a simple, highly sensitive, fast, and versatile naked-eye detection methodology that could be coupled to any LAMP or RT-LAMP assay, avoiding the need of using complicated sample pretreatments and/or AuNPs long and laborious functionalization processes.

Loop-mediated isothermal amplification (LAMP) has become a powerful alternative to polymerase chain reaction (PCR) for pathogen detection in clinical specimens^1,2^ and food matrices.^3,4^ Owing to the ability of Bst DNA polymerase to display a high strand displacement activity at a constant temperature (60–65 °C), isothermal LAMP has the potential to revolutionize molecular biology using simpler instrumentation, as well as faster and more efficient assays than PCR, even for crude samples.^5^ Consequently, LAMP has been adopted as a low-technology molecular analysis tool for resource-limiting areas and point-of-care (POC) applications.^6^ Achieving efficient detection at the POC, though, depends largely on the method selected for amplicon detection.

Naked-eye monitoring is the most promising method for inexpensive and simple diagnostics, with colorimetric detection being a prevalent approach for POC applications.^7^ The most common way to induce a nucleic acid-dependent colorimetric change is to use pH-sensitive dyes by directly integrating them into a LAMP reaction;^8,9^ this is key in order to reduce the risk of contamination and provides a simple color discrimination between the positive and negative samples. The potential drawbacks of using pH-sensitive dyes are the inability of the assay to generate sufficient pH variation, as reported for some targets and sample types,^10^ and the need for a weakly buffered solution that restricts their use in the LAMP reaction.^11^ Another commonly used approach is the use of hydroxy naphthol blue (HNB) metal indicator dye, where by taking advantage of the synergistic effect of LAMP’s byproducts (pyrophosphate-PPi^4–^ and hydrogen-H^+^ ions), an improved sensitivity compared to pH-sensitive dyes in weakly buffered solutions has been reported.^12^ Such methods, however, may exhibit false-positive results derived from nonspecific amplification; an HRP-mimicking molecular beacon has been reported to correct false-positives, but this approach increases the assay’s complexity overall due to the need for specific beacon designs.^13^

An alternative to the above-mentioned dyes is metallic nanoparticles (NPs), which can exhibit a size- and/or shape-dependent surface plasmon resonance (SPR) in the visible spectrum.^14^ Gold NPs (AuNPs), used extensively due to their strong quantum size effect and high stability, are capable of generating naked-eye visible color changes (e.g., from red to blue/purple, red to pink, and contrariwise)^15,16^ as a result of the NPs stabilization (dispersed) or destabilization (aggregated) induced by the presence or absence (or vice versa) of the target. There are two main approaches for AuNPs-based visual detection of amplicons: the target-specific and target-independent methods.^7^ The former involves AuNPs functionalized with oligonucleotide (OG) probes, where the state of the AuNPs relies on the complementarity of the probe with the target nucleic acid. The target-specific method has been used in combination with LAMP assays to detect different pathogens, with reported detection limits within the range of 10–200 copies per reaction;^17,18^ however, this was achieved upon DNA extraction, which is still a lab-based methodology. Recently, AuNPs-based target-specific assays have been developed in combination with the trans-cleavage activity of CRISPR/Cas systems, providing distinct colorimetric differences between the positive and negative samples, either by reverting or by promoting AuNPs aggregation.^19−21^ Notable drawbacks of the target-specific naked-eye colorimetric assays are the need for laborious and time-consuming protocols, including the need for the AuNPs surface-functionalization and/or multiple sample manipulation steps, e.g., mixing the amplified product with the AuNP probes, incubation, and later reopening for salt addition, resulting in a contamination risk.

The target-independent detection method primarily relies on the stabilization or destabilization of AuNPs owing to electrostatic interactions. When AuNPs were coated with 11-mercaptoundecanoic acid (MUA), they aggregated in the presence of magnesium ions (Mg^2+^) in a negative sample, while they were stabilized when magnesium pyrophosphate (Mg_2_P_2_O_7_), one of the LAMP byproducts, was formed in a positive sample (red solution).^22^ Furthermore, when AuNPs were cofunctionalized with MUA and polyethylene-glycol (PEG), they were stable in the presence of Mg^2+^ in a negative sample due to steric hindrance (red solution), while they aggregated in the presence of Mg_2_P_2_O_7_ in a positive sample (red precipitate).^23^ The aforementioned assays have been demonstrated for the purified samples of extracted DNA with reported detection limits of 200 and 500 copies per reaction, respectively. An advantage of these assays is the ability to incorporate the AuNPs in the LAMP solution, although sonication is occasionally necessary to prevent aggregation; disadvantages include the dependency on the Mg_2_P_2_O_7_ byproduct and the requirement of AuNPs surface functionalization.

Cationic polymers provide another potential means for target-independent detection of amplified DNA via electrostatic interactions. An early study^24^ demonstrated this concept when positively charged polyethylenimine (PEI) was added to the LAMP reaction with a fluorescent-labeled OG probe, allowing visualization down to 0.2 μg of lambda DNA target in a purified sample. The main drawbacks of the method are the increased contamination risk because of the addition of PEI after LAMP and the requirement of a UV illuminator and fluorescent probes for optical detection. In another study,^25^ chitosan (Chit) polysaccharides together with AuNPs were used for the naked-eye detection of *Mycobacterium tuberculosis* using as a starting material ∼30–400 μg/mL of whole DNA extracted from sputum. However, this assay was demonstrated in combination with PCR, a lab-based method using purified DNA; thus, it is unsuitable for POC applications.

Chitosan, a polysaccharide derivative of chitin sourced from the seafood industry, is a promising polymer because its cationic form (pH < p*K*_a_ of ∼6.5)^26^ can conjugate DNA electrostatically, forming a Chit-DNA complex.^27,28^ The use of Chit-conjugated AuNPs has been reported in various applications, such as biosensing,^29^ drug delivery,^30^ and tumor targeting,^31^ among others. Furthermore, Chit-coated magnetic NPs have been extensively used to extract DNA in acidic environments in which their charge is positive and then release it in more basic environments in which their charge gets neutralized.^32−34^ A main advantage of Chit-capped AuNPs is the ability to synthesize them using a green synthetic procedure. Chitosan is a nontoxic, eco-friendly, biosafe, and biodegradable material.^35^ Moreover, it has been demonstrated to act both as a reducing and stabilizing agent in a simple AuNPs synthesis procedure based on chemical reduction in aqueous environments;^36^ this method does not require toxic solvents or extra reducing agents. Although Chit appears promising for DNA-binding combined with aggregation/stabilization and colorimetric detection, its compatibility with LAMP has been low.^37^ To the best of our knowledge, Chit-capped AuNPs visual observation of LAMP products has not yet been reported. Overall, despite recent advances, the development of simple and economic colorimetric assays for naked-eye detection with high sensitivity remains a challenge.

Herein, we report the development of a simple, fast, and cost-effective method for naked-eye colorimetric detection of amplified nucleic acids produced via LAMP using synthesized Chit-AuNPs and *Salmonella typhimurium* as the selected target for demonstrating the proof-of-principle. Using a facile, rapid green synthesis protocol, positively charged Chit-AuNPs were prepared and directly used for amplified DNA detection without further modifications. To overcome the incompatibility of the synthesized Chit-AuNPs solutions with the LAMP reaction, we immobilized appropriate amounts of Chit-AuNPs inside the lid of the tubes by surface tension, which was mixed with the reaction after the amplification process was completed. Using the above-mentioned one-tube assay, we avoided possible aerosol contamination by eliminating the need to open the tube and developed an end-point colorimetric assay with a time-to-result of ∼35 min. Based on the presence or absence of free Chit in the Chit-AuNPs suspension, we demonstrated two colorimetric detection approaches, both of which could be used in the presence of crude saliva samples. Moreover, we showed that both methods can be used for the efficient naked-eye detection of the *Salmonella* target in the range of 5–1000 cfu/reaction, while the assay with the optimized free Chit exhibited an impressive detection limit of 1 cfu/reaction in both pure and crude saliva samples. In addition to the detection of the bacterial *Salmonella* target, the SARS-CoV-2 target was used as a proof of the method’s generic applicability toward detecting viral targets in combination with reverse transcriptase LAMP (RT-LAMP), giving a detection limit of 10 copies/reaction.

## Experimental Section

### Reagents and Materials

Gold(III) chloride trihydrate (HAuCl_4_·3H_2_O, ACS reagent, ≥49% Au basis), chitosan low molecular weight (LMW, 50–190 kDa, 75–85% deacetylated), acetic acid (glacial, ACS reagent, ≥99.7%), water for chromatography (LC–MS grade) LiChrosolv, and phosphate buffer saline (PBS) tablets were purchased from Merck (Darmstadt, Germany). All chemicals were used as received without any further purification. The set of six primers (100 μM) for the *Salmonella* invasion gene *invA* was purchased from Metabion (Germany), while six primers for the SARS-CoV-2 detection targeting the N gene were purchased from Eurofins Genomics (Germany). Warmstart Multi-Purpose LAMP/RT-LAMP 2× Master Mix, WarmStart Colorimetric LAMP 2× Master Mix (DNA & RNA), and LAMP fluorescent dye (readable in the SYBR/FAM channel of real-time fluorimeters) were purchased from New England BioLabs. Normal saliva (pooled from human donors) was purchased Lee Biosolutions, USA. Mineral oil (BioReagent, for molecular biology) was purchased from Merck. Synthetic SARS-CoV-2 RNA was purchased from BIORAD (SARS-CoV-2 Standard #COV019 and SARS-CoV-2 Negative #COV000).

### Synthesis of AuNPs Capped with Chitosan

Chit-AuNPs were synthesized utilizing a chemical reduction process, with chitosan acting as both a reducing and stabilizing agent.^38^ Volumes of 15 mL of 0.15, 0.25, and 0.35% (w/v) LMW chitosan solutions [in 1% (v/v) aqueous acetic acid] were heated to a temperature of 45 °C and stirred for 3 h. Afterward, the temperature was raised to 75 °C, and 0.15 mL of an aqueous HAuCl_4_ solution (100 mM) was added to each of the solutions while under stirring. After 2 h of vigorous stirring under heating, the solutions were moved away from the heating plate and remained under stirring until room temperature (*T*_R_). The solutions were then stored at 4 °C for further use. In all cases, different shades of red were obtained, indicating the formation of Chit-AuNPs in different sizes/concentrations. The color change from light yellow to red started in the solutions with higher Chit concentrations first, which means that the reduction process was faster.

### Colorimetric LAMP Assay Preparation and Evaluation

Evaluation experiments were performed with an attenuated strain of *S. typhimurium* as the target. *Salmonella* was grown overnight in Luria–Bertani (LB) medium, and the cultures were subsequently measured spectrophotometrically at an OD_600_ of 0.23 corresponding to a cell concentration of 3 × 10^8^ cfu/mL. Cell lysis (heating at 95 °C for 10 min) was performed in the *Salmonella* cells stock solution before further use, while serial dilutions using PBS were carried out to reach the final required concentrations. A PBS solution (0.01 mol/L, pH 7.4) was prepared by dissolving a PBS tablet in 1 L of ultrapure water. For the purified samples, the LAMP reagents mix in a total volume of 25 μL contained 12.5 μL of Warmstart, 2.5 μL of primer mix (containing 18 μM FIP and BIP, 2 μM F3 and B3, and 6 μM Loop-F and Loop-B), 9 μL of nuclease-free water, and 1 μL of the target at appropriate dilutions (as a negative control, 1 μL of PBS solution was used). For the crude saliva samples, the LAMP reagents mix was similar, but 2.5 and 5 μL of lysed saliva (95 °C for 10 min) replaced the corresponding amount of nuclease-free water in the final reaction’s volume. A cross-study in the RT-LAMP assay using SARS-CoV-2 as the target was carried out with the corresponding reagents amounts similar to the LAMP-*Salmonella* crude saliva sample assays. In this case, the dilutions from the target’s starting stock to different SARS-CoV-2 copies were performed in nuclease-free water (as a negative control, 1 μL of neat nuclease-free water was used). Volumes of 7.5 μL of Chit-AuNPs solutions were immobilized inside the tube’s lid by surface tension in order to not interfere with the LAMP reagents, as well as to avoid aerosol contamination. After the LAMP reaction was complete (at *T* = 63 °C for *t* = 30 min in the *Salmonella* assay and *T* = 65 °C for 30 min in the SARS-CoV-2 assay), a brief spin-down (∼10 s) led to the mixture of the two different solutions, and the result was ready to be read colorimetrically by the naked eye. For the Chit-AuNPs-based assays, heating of the 0.2 mL PCR tubes (Sarstedt) was performed using a FastGene Ultra Cycler Gradient (Nippon GENETICS Europe), where no heating on top of the tubes was applied as it could evaporate the immobilized Chit-AuNPs solutions. Real-time quantitative colorimetric LAMP (qcLAMP) using phenol red (pH indicator) was performed using Pebble (Biopix-T, Gr),^39^ where 15 μL of mineral oil was added over the LAMP mix, in order to avoid solvent evaporation owing to the tubes’ heating. Finally, a Coyote Mini8 Plus real-time PCR cycler (Coyote Bioscience Co., Ltd., Beijing, China) was used for the real-time fluorescent LAMP assays.

## Results and Discussion

### Synthesis and Characterization of Chit-AuNPs

Detailed description of the Chit-AuNPs synthesis and characterization can be found in Supporting Information. Specifically, a schematic representation of the synthesis reaction is shown in Figure S1. Regarding the characterization, attenuated total reflectance-Fourier transform infrared (ATR-FTIR) spectroscopy confirmed the presence of chitosan on the synthesized AuNPs (Figure S2a), while UV–Vis spectroscopy revealed their plasmonic behavior (Figure S2b). Dynamic light scattering (DLS) measurements provided information about the hydrodynamic size and polydispersity of the colloidal solutions, while zeta potential (ZP) values further confirmed the presence of cationic chitosan on the AuNPs surface (Figure S2c). Finally, scanning electron microscopy (SEM) images were used to study the size and morphology of the synthesized Chit-AuNPs (Figure S3).

### Naked-Eye Detection of LAMP Amplicons in Purified Samples Using Chit-AuNPs

A previous study has shown that directly incorporating water-soluble Chit in a LAMP reaction inhibits DNA amplification because positively charged Chit conjugates with negatively charged DNA or interferes with LAMP primer annealing.^37^ This was further confirmed when we added either dissolved neat Chit or Chit-AuNPs in their originally synthesized acidic environment (replaced 5 μL of nuclease-free water with Chit or Chit-AuNPs) and included as well 0.5 μL of LAMP fluorescent dye (readable in the SYBR/FAM channel) for detection; in both cases, no amplification signal was observed (Figure S4) as the pH of the final mixture (measured with a pH strip) is ∼4.5 owing to the acetic acid addition.

**Figure 1:**
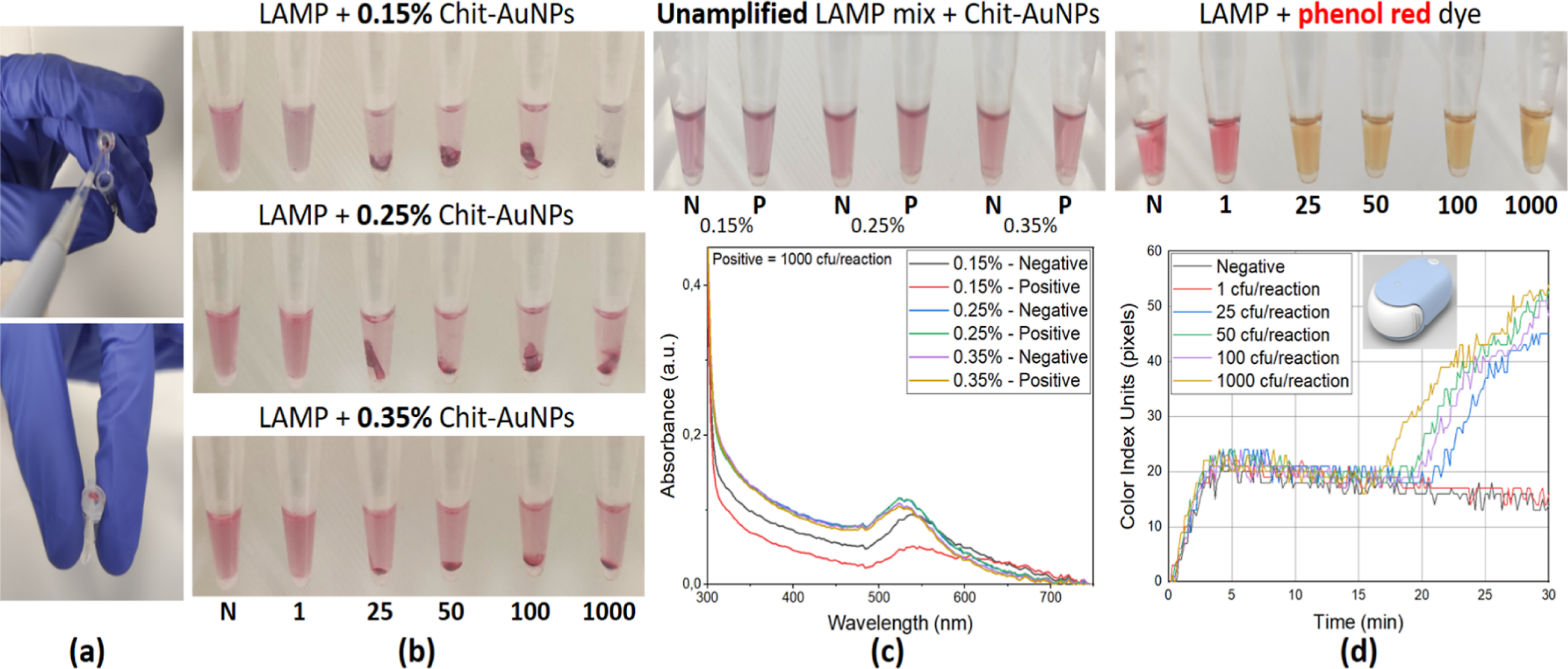
(a) Immobilizing appropriate amounts of Chit-AuNPs solutions inside the lids of the tubes. (b) End-point colorimetric results in purified samples using the Chit-AuNPs synthesized with the different Chit concentrations; pellet formation and supernatant discoloration can be observed with increasing number of target cfu/reaction. (c) Photograph of the tubes and UV–Vis absorbance spectra of the unamplified LAMP reaction mixed with the different Chit-AuNP solutions. (d) Photograph of the tubes and real-time qcLAMP diagram (via Pebble) using phenol red. Note: the values of 1–1000 below the photographs of the 0.2 mL tubes correspond to *Salmonella* target concentration in cfu/reaction, N corresponds to negative samples, and P corresponds to positives with 1000 cfu/reaction. Experiments were performed in triplicate.

Based on the above, we developed a single-tube assay where 7.5 μL of the synthesized colloidal Chit-AuNPs solutions including free dissolved nonreacted Chit, was immobilized inside the lids of the tubes (Figure 1a). This approach was used to test all three different-sized Chit-AuNPs, employing the *SalmonellaInvA* gene as the target, within a concentration range of 1–1000 cfu/reaction. After the LAMP reactions were completed (30 min), a brief spin-down (∼10 s) of the tubes was carried out in order to mix the solutions.

According to Figure 1b, in all cases [0.15, 0.25, and 0.35% (w/v) Chit-AuNPs], the 25 cfu/reaction *Salmonella* concentration was detected based on pellet creation and supernatant discoloration, while the negative samples were stable. Moreover, in the Chit-AuNPs synthesized with the lowest Chit concentration (0.15%, w/v), a slight color change was observed even at 1 cfu/reaction *Salmonella* concentration. The higher sensitivity observed for the 0.15% (w/v) Chit-AuNPs was further confirmed by directly adding the three different Chit-AuNPs solutions inside a LAMP reaction mixture with 1000 cfu/reaction (positive) or without (negative) target DNA and observing the color change without amplification. As shown in Figure 1c, for the 0.15% (w/v) Chit-AuNPs, a color change was detected by the naked eye between the negative and the positive samples, along with a drop in the UV–Vis absorbance intensity in the case of the positive sample. In the case of the Chit-AuNPs synthesized with the higher Chit concentrations, both the negative and positive samples retained the same color and absorbance intensities.

Parallel to the above-mentioned end-point naked-eye colorimetric detection using Chit-AuNPs, colorimetric LAMP using phenol red was used to further evaluate the assay. For these experiments, we used a real-time colorimetric device that can perform quantitative colorimetric LAMP (qcLAMP).^39^ As shown in Figure 1d, we could also detect *Salmonella* concentrations as low as 25 cfu/reaction using this method but not 1 cfu/reaction. The time-to-positive result was ∼17 min for the 1000 cfu/reaction, ∼19.5 min for the 100 and 50 cfu/reaction, and ∼21 min for the 25 cfu/reaction. These results confirm that the Chit-AuNPs colorimetric detection methodology is equally or more sensitive to a colorimetric real-time method, although the Chit-AuNPs approach cannot be used for real-time quantification.

### Naked-Eye Detection of LAMP Amplicons in Saliva Samples Using Chit-AuNPs

**Figure 2:**
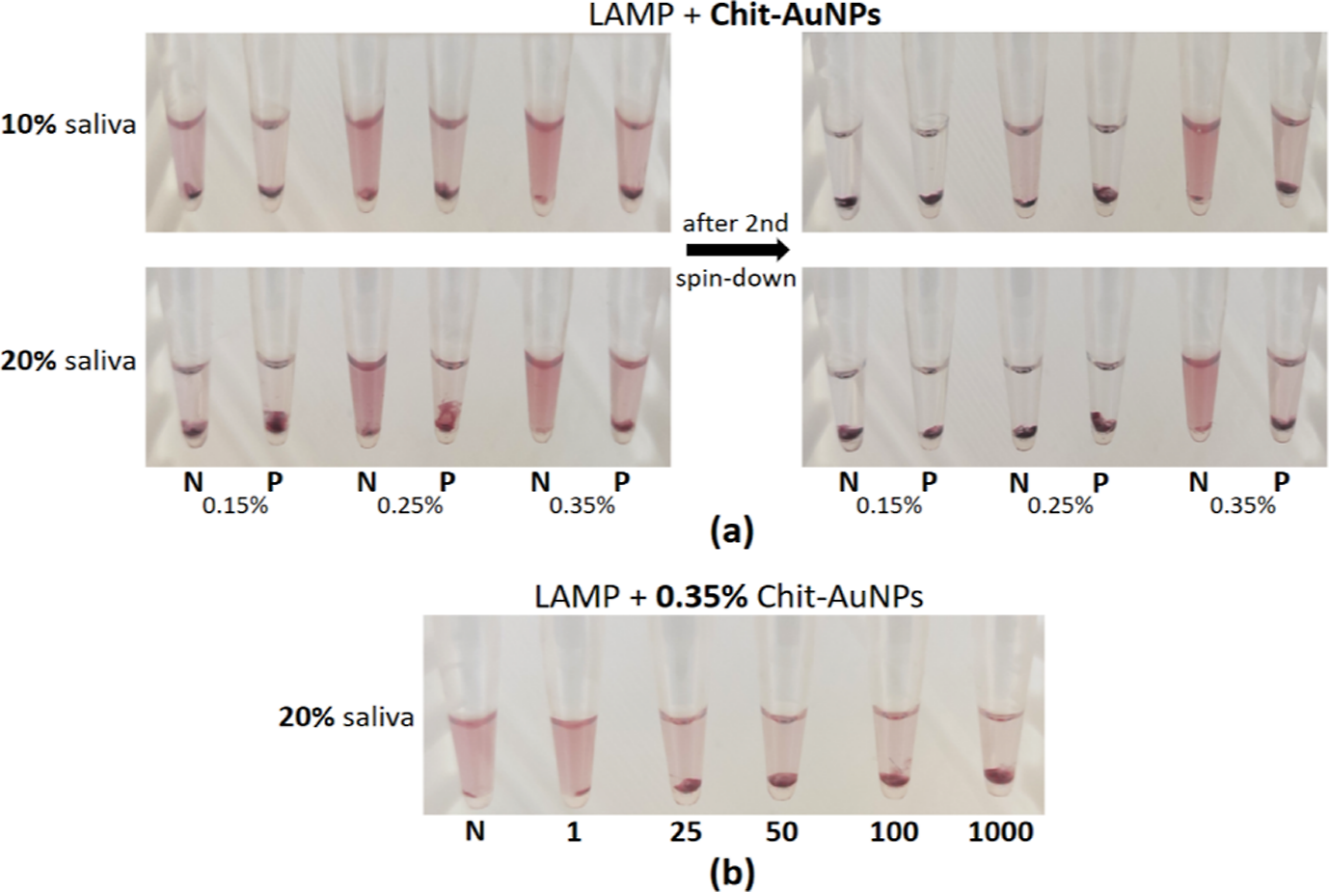
(a) Observation of the different Chit-AuNPs stabilization efficiencies on the negative samples in the presence of different amounts of lysed saliva samples inside the LAMP mix. (b) End-point colorimetric results using the synthesized 0.35% (w/v) Chit-AuNPs, in different target cfu/reaction, in a LAMP mix containing 5 μL of saliva (20%). Note: the values of 1–1000 below the 0.2 mL tubes correspond to *Salmonella* target concentration in cfu/reaction, N corresponds to negative samples, and P corresponds to positives with 100 cfu/reaction. Experiments were performed in triplicate.

Experiments were also conducted with crude saliva samples to further test the applicability of the Chit-AuNPs-based colorimetric assay for POC applications. Initially, we tested the effect of saliva on the stability of the Chit-AuNPs in the LAMP reaction by testing one positive sample, containing 100 cfu/reaction of the *Salmonella* target, and one negative sample. In particular, we replaced 2.5 or 5 μL of the nuclease-free water in the LAMP mix with lysed saliva. These amounts correspond to a final 10 and 20% saliva sample concentration, respectively, one of the highest percentages of crude samples in a LAMP reported so far. At the same time, similar amounts (7.5 μL) of the three Chit-AuNPs solutions were immobilized inside the lids of the tubes. After the LAMP reactions were completed, a brief spin-down of the tubes was conducted to mix the solutions and help reveal the changes in the color fast. A pellet was observed in the negative samples, primarily at the two lower concentrations (0.15 and 0.25%, w/v), along with an intense discoloration of these two solutions (Figure 2a, left). After a second spin-down, the only solution that retained its color was the negative sample with 0.35% (w/v) Chit-AuNPs (Figure 2a, right) for both 2.5 and 5 μL saliva samples, while the discoloration of the negatives (0.15 and 0.25%, w/v) was stronger and faster in the 5 μL (20%) than in the 2.5 μL (10%) saliva sample. The positive samples containing 100 cfu/reaction of the target presented aggregation in all cases, as observed before with pure samples (Figure 1b). Out of these results, it can be concluded that in the negative samples, the free Chit was not sufficient to efficiently protect the 0.15 and 0.25% (w/v) Chit-AuNPs from aggregation induced by the different saliva components. However, we cannot exclude the effect of the three different Chit-AuNPs in the above behavior because of the different Chit-AuNPs sizes and concentrations.

The solution of 0.35% (w/v) Chit-AuNPs, the most efficient to maintain Chit-AuNPs stabilization in the presence of saliva, was further used for the clear naked-eye detection of different target amounts within the range of 25–1000 cfu/reaction of the *Salmonella* target (Figure 2b).

To further evaluate the effect of free Chit and the final solution’s pH on differentiating negative and positive samples, 0.5 mL of 0.15% (w/v) Chit-AuNPs solution was centrifuged as it had the worst performance in the saliva samples. Afterward, the supernatant containing the free Chit was removed, and the Chit-AuNPs pellets were redispersed in three solutions: (a) 1% (v/v) aqueous acetic acid; (b) ultrapure water; and (c) 1% (v/v) aqueous acetic acid containing 0.15% (w/v) Chit. The resulting solutions showed no aggregation after the purification step and redispersion in the different media (Figure S5).

**Figure 3:**
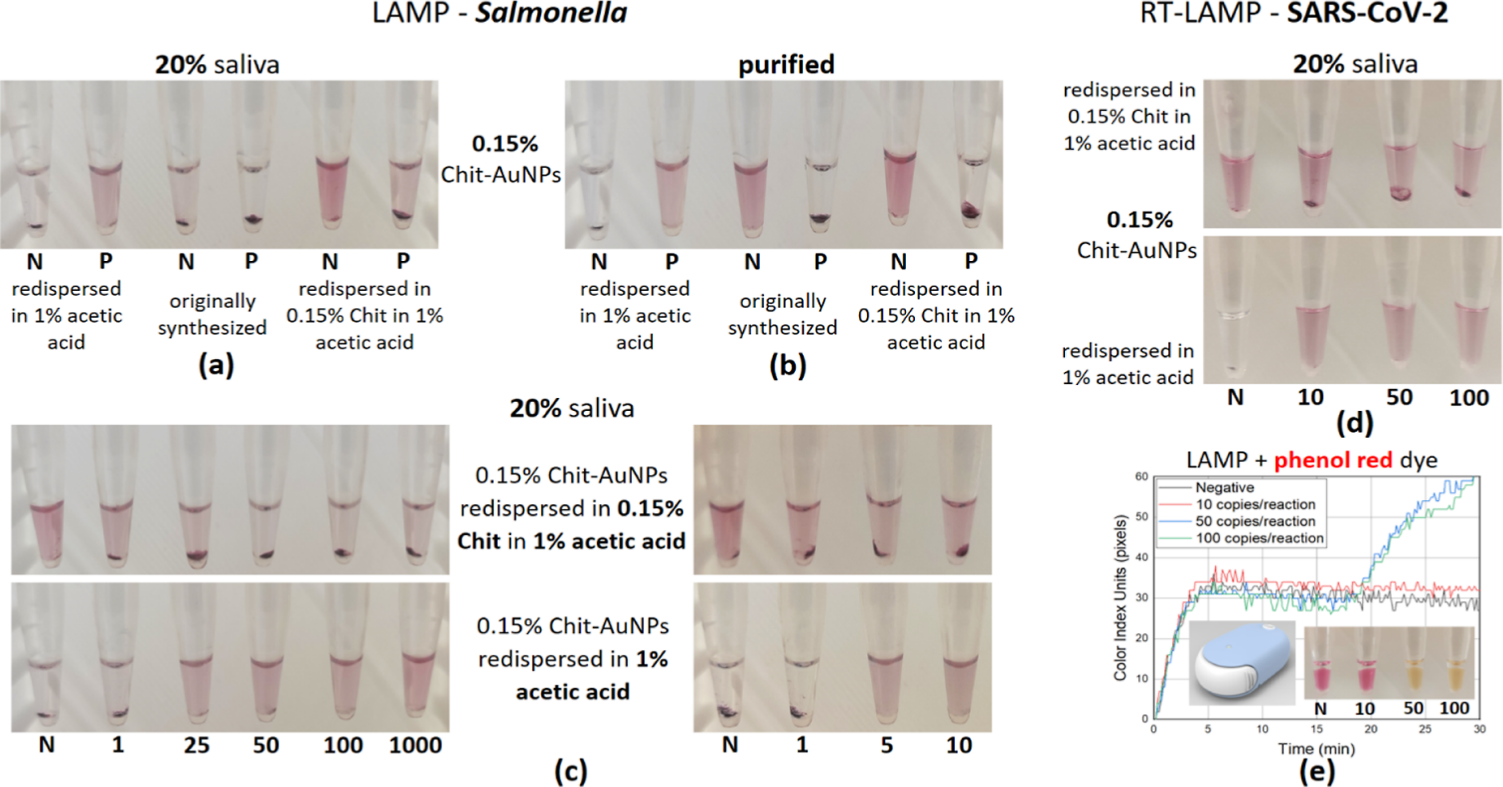
(a,b) Comparative colorimetric results between the initially synthesized 0.15% (w/v) Chit-AuNPs and the redispersed Chit-AuNPs in either 1% (v/v) aqueous acetic acid or 0.15% (w/v) Chit in 1% (v/v) aqueous acetic acid for both purified and crude saliva samples (20%). (c) End-point colorimetric results using the redispersed Chit-AuNPs—mirror-like results based on the presence or absence of free Chit in the solution for 20% saliva samples (*Salmonella* target). (d,e) Photographs of end-point colorimetric detection and a real-time qcLAMP diagram (via Pebble), after an RT-LAMP reaction containing 20% saliva samples (SARS-CoV-2 target). Note: the values of 1–1000 below the 0.2 mL tubes correspond to target cfu (*Salmonella*) or copies (SARS-CoV-2)/reaction, N corresponds to negative samples, and P to positives with 100 cfu/reaction. Experiments were performed in triplicate.

As seen before, the initially synthesized 0.15% (w/v) Chit-AuNPs were not able to provide a distinct colorimetric difference between the positive (100 cfu/reaction) and negative samples after the LAMP reaction in 20% saliva because Chit-AuNPs aggregation occurred in both solutions (Figure 3a, middle). The addition of excess Chit in the solution (0.15%, w/v) was able to inhibit the complete Chit-AuNPs aggregation of negative samples (Figure 3a, right), similar to the 0.35% (w/v) Chit-AuNPs solution in the crude saliva samples, further confirming the role of free Chit itself in the solution. Interestingly, the Chit-AuNPs solution without any free Chit, i.e., the one redispersed in 1% (v/v) aqueous acetic acid, provided the opposite results, with aggregation in the negative sample, and the positive ones remained stable (Figure 3a, left). When we repeated these experiments in pure samples, a distinct colorimetric difference was observed in all cases (Figure 3b).

Moreover, the two 0.15% (w/v) Chit-AuNPs solutions, with or without excess free Chit in 1% (v/v) aqueous acetic acid, were tested with different amounts of the target within the range of 1–1000 cfu/reaction (Figure 3c, left) for the saliva samples. In both cases, the 25 cfu/reaction target concentration was easily detected colorimetrically by the naked eye in the LAMP reaction, while the 1 cfu/reaction target concentration was detected only when the Chit-AuNPs were redispersed in excess Chit (0.15%, w/v). Testing closer, the efficiency of the two assays in the range of 1–25 cfu/reaction confirmed the above-mentioned observations; notably, the 1 cfu/reaction target concentration was detected in half of the tested samples (number of samples: 6) (Figure 3c, right), probably reflecting the probability of capturing the target with every pipetted sampling.

In a follow-up step, RT-LAMP experiments further confirmed that the aforementioned method could also detect SARS-CoV-2 in the presence of 20% saliva with a detection limit of 10 copies/reaction (Figure 3d). This result proves that the assay is flexible and generic as it can be used with different targets and in combination with RT. Comparison with colorimetric detection using phenol red (qcLAMP) further confirmed the higher sensitivity of the Chit-AuNPs detection method, compared to pH-sensitive dyes (Figure 3e). The time-to-positive was ∼19 min for both 100 and 50 cfu/reaction target concentrations in the phenol red system, while the 10 copies/reaction were not able to be detected, probably due to insufficient H^+^ byproducts produced during amplification that shall induce a significant pH drop and/or due to the possibly increased buffer capacity and pH in the presence of the crude (5 μL saliva) sample. Finally, the high specificity of both the *Salmonella* DNA and SARS-CoV-2 RNA amplification reactions was demonstrated during the detection of zero false positives in all the no-template reactions tested (>30) within the 30 min of the assay.

### Mechanism of the Chit-AuNP-Based Colorimetric Detection of LAMP Amplicons

To explain the above-mentioned results, we investigated both the ability of Chit to exist in a pH-dependent charged form and the mechanism behind AuNPs stabilization. For a p*K*_a_ value of ∼6.5, Chit is protonated at a lower pH and exists in a cationic form and a DNA-binding state. AuNPs can be stabilized and remain dispersed in a solution because of electrostatic repulsive forces. Additionally, when polymer molecules are attached to the AuNPs, forming a coating, steric stabilization is achieved via repulsive forces that separate the particles from one another, while depletion stabilization can also be observed in the presence of free polymeric molecules in the solution. The combination of all three colloidal stabilization factors is shown to provide ultrahigh NPs stability under different conditions,^40,41^ which would otherwise lead to aggregation. Herein, different destabilization agents (salt, nontarget DNA, dNTPs, and other elements in the saliva) in the LAMP mix can lead to NPs aggregation.

**Figure 4:**
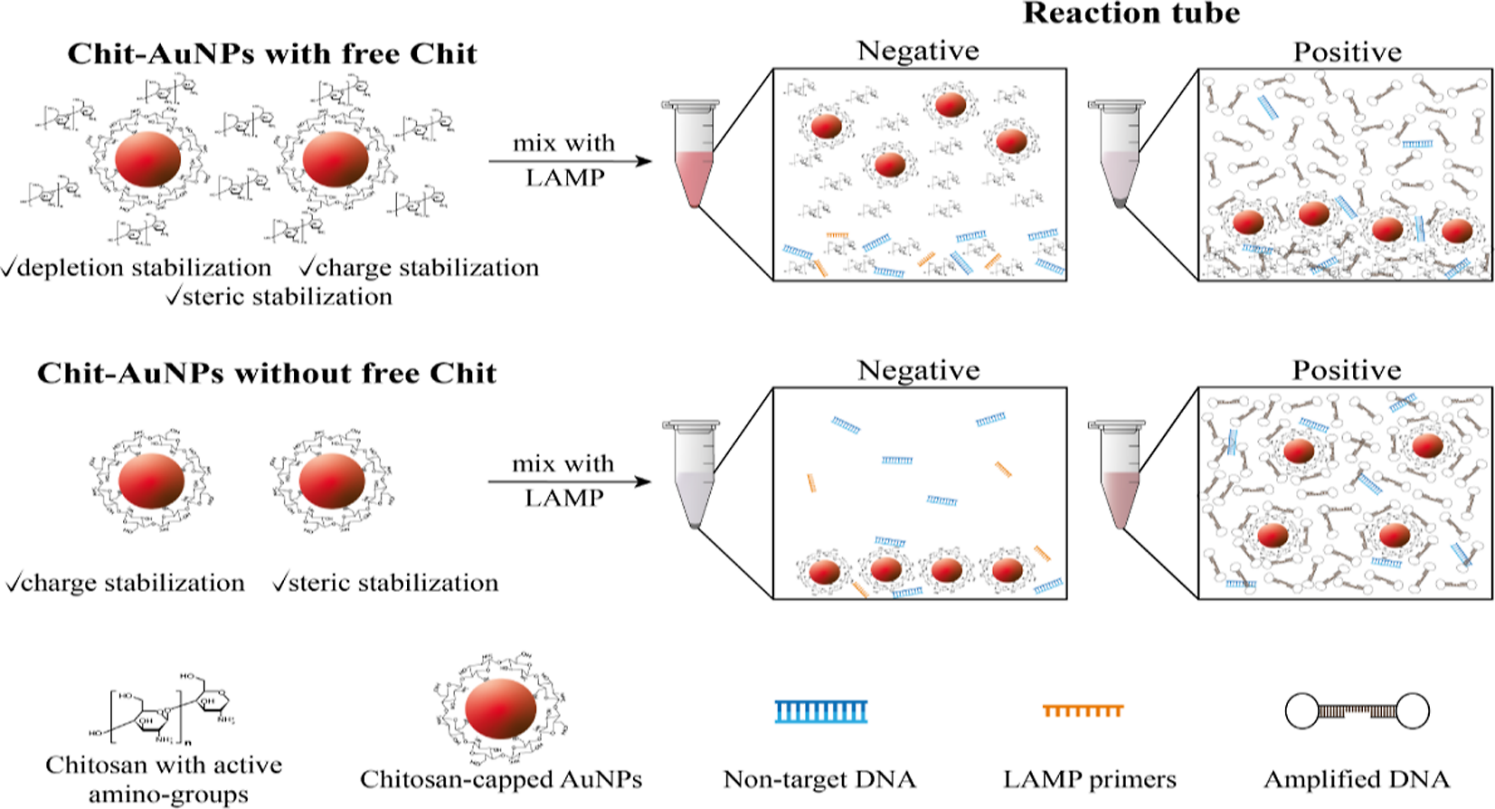
Close-up image of the Chit-AuNPs, free Chit, and (amplified) DNA interactions inside the LAMP mix after amplification reaction. Note that the Chit-AuNPs solutions are dissolved in 1% (v/v) aqueous acetic acid in both cases.

Our results indicate that when using Chit-AuNPs with free Chit in purified negative samples, the free Chit polymer can protect the Chit-AuNPs from aggregation induced by the different LAMP reagents, keeping the AuNPs dispersed in the solution owing to the depletion stabilization effects. However, it appears that there is an optimum free Chit amount required for stabilization depending on the sample type (purified vs saliva). Although the free Chit in the 0.15% (w/v) synthesized Chit-AuNPs is sufficient to provide stabilization in purified negative samples (Figure 3b, middle), it is not equally effective in crude negative samples (Figure 3a, middle). In saliva, this amount was insufficient to cope with the extra DNA released upon cell lysis and possible variations in the pH and salt;^42,43^ hence, a higher amount of free Chit concentration is essential to induce the same result. In both pure and saliva-containing positive samples, the high yields of negatively charged DNA amplicons combined with the positively charged free Chit and Chit-AuNPs (in the acidic final solution of ∼4.5 pH) disturb stabilization via electrostatic interactions, causing Chit-AuNPs aggregation. However, in the presence of saliva, the negative samples also tend to aggregate after ∼4 days, possibly due to the differently charged antagonistic populations inside the solution (Figure S6a). A schematic diagram of the Chit-AuNPs stabilization mechanism with free Chit is depicted in Figure 4, top line.

Conversely, when using Chit-AuNPs without free Chit in the solution, a “mirror effect” is observed; the negative samples are destabilized in the LAMP mix even in the presence of 20% saliva, with the Chit-AuNPs aggregating due to the absence of the free Chit depletion protection. At the same time, the positive samples remain stable as large amounts of DNA amplicons can electrostatically coat the positively charged Chit-AuNPs, protecting them from aggregation via repulsion forces (Figure 4, bottom line). The complete coating/stabilization of the positively charged Chit-AuNPs with negatively charged amplified DNA in the stable positive samples was also confirmed by the z-potential and UV–Vis measurements (Figure S7a,b). The low pH of ∼4.5 in the final solution [LAMP mix + Chit-AuNPs redispersed in 1% (v/v) aqueous acetic acid] is essential for the electrostatic stabilization of the positive samples; indeed, when the same experiment was repeated with Chit-AuNPs redispersed in ultrapure water, the positive samples also aggregated (Figure S6b). Notably, the concentration of Chit-AuNPs used in this method is also crucial as low concentrations of NPs may lead to stabilization by the nontarget DNA contained in the saliva, eliminating the possibility of differentiating the negative samples from the positive ones (false positives).

## Conclusions

Herein, we exploited the effect of free Chit in the synthesized Chit-AuNPs solutions to create two different naked-eye end-point colorimetric assays combined with LAMP. Using the different stabilization forces (depletion, electrostatic, and steric) between the pH-responsive Chit and Chit-AuNPs, we developed two mirror-like assays, one in the presence and the other in the absence of free Chit. In both cases, we demonstrated the ultrasensitive eye-detection of the *Salmonella* target with a detection limit of 1 cfu/reaction (40 cfu/mL) in the presence of free Chit and 5 cfu/reaction (200 cfu/mL) in the absence of free Chit in the Chit-AuNPs solution. The above performance was also shown in an impressive final saliva concentration of 20%. The general applicability of the method toward the detection of viral targets was also demonstrated when 10 copies/reaction (400 copies/mL) of SARS-CoV-2 in 20% saliva, amplified with RT-LAMP, were successfully detected by the naked eye. This colorimetric detection method combines many attractive features, such as simplicity, high sensitivity, and rapid results, making it an ideal candidate for incorporation into POC applications. Using a “green” synthesis of Chit-AuNPs is considered an added advantage. Being a target-independent method, this assay does not provide any added degree of specificity, which entirely depends on the selected primers. However, this limitation leads to one advantage of this method, which is compatibility with any kind of (RT-)LAMP assay. Currently, both assays rely on end-point detection; however, the assay using free Chit and Chit-AuNPs could be employed for quantitative results based on the decrease of the absorbance intensity of the supernatant (discoloration), when the final solutions are measured at the same time. Such a quantification method could be more sensitive than colorimetric assays using pH-sensitive dyes because the free Chit/Chit-AuNPs system essentially detects DNA under appropriate pH conditions and not LAMP-induced pH changes. Finally, this assay could possibly be applied to other isothermal amplification methods, such as (RT-)RPA, where amplification happens at even lower temperatures (37–42 °C), providing another efficient, fast, sensitive, and cost-effective POC system.

## Supporting Information Available

LAMP primer sequences for *Salmonella* invasion gene invA and SARS-CoV-2; Chit-AuNPs purification via centrifugation; Chit-AuNPs characterization; Chit-AuNPs molar concentration calculation; schematic representation of the Chit-AuNPs synthesis reaction; spectroscopic and morphological characterization of the synthesized Chit-AuNPs; LAMP reaction in the presence of Chit-AuNPs; purification of Chit-AuNPs via centrifugation; effect of time and pH on the stability of the final solutions; and zeta potential and UV–Vis absorbance measurements of the final stable solutions (PDF)

## References

[ref1] Heithoff D. M.; Barnes L.; Mahan S. P.; Fox G. N.; Arn K. E.; Ettinger S. J.; Bishop A. M.; Fitzgibbons L. N.; Fried J. C.; Low D. A.; Samuel C. E.; Mahan M. J. Assessment of a Smartphone-Based Loop-Mediated Isothermal Amplification Assay for Detection of SARS-CoV-2 and Influenza Viruses. JAMA Netw. Open 2022, 5 (1), e2145669 10.1001/jamanetworkopen.2021.45669. 35089353 PMC8800074

[ref2] Garg N.; Sahu U.; Kar S.; Ahmad F. J. Development of a Loop-Mediated Isothermal Amplification (LAMP) Technique for Specific and Early Detection of Mycobacterium Leprae in Clinical Samples. Sci. Rep. 2021, 11 (1), 9859 10.1038/s41598-021-89304-2. 33972644 PMC8110778

[ref3] Narushima J.; Kimata S.; Soga K.; Sugano Y.; Kishine M.; Takabatake R.; Mano J.; Kitta K.; Kanamaru S.; Shirakawa N.; Kondo K.; Nakamura K. Rapid DNA Template Preparation Directly from a Rice Sample without Purification for Loop-Mediated Isothermal Amplification (LAMP) of Rice Genes. Biosci., Biotechnol., Biochem. 2020, 84 (4), 670–677. 10.1080/09168451.2019.1701406. 31842715

[ref4] Kaur A.; Kapil A.; Elangovan R.; Jha S.; Kalyanasundaram D. Highly-Sensitive Detection of Salmonella Typhi in Clinical Blood Samples by Magnetic Nanoparticle-Based Enrichment and in-Situ Measurement of Isothermal Amplification of Nucleic Acids. PLoS One 2018, 13 (3), e0194817 10.1371/journal.pone.0194817. 29590194 PMC5874042

[ref5] Österdahl M. F.; Lee K. A.; Lochlainn M. N.; Wilson S.; Douthwaite S.; Horsfall R.; Sheedy A.; Goldenberg S. D.; Stanley C. J.; Spector T. D.; Steves C. J. Detecting SARS-CoV-2 at Point of Care: Preliminary Data Comparing Loop-Mediated Isothermal Amplification (LAMP) to Polymerase Chain Reaction (PCR). BMC Infect. Dis. 2020, 20 (1), 783 10.1186/s12879-020-05484-8. 33081710 PMC7574392

[ref6] Das D.; Lin C.-W.; Chuang H.-S. LAMP-Based Point-of-Care Biosensors for Rapid Pathogen Detection. Biosensors 2022, 12 (12), 1068 10.3390/bios12121068. 36551035 PMC9775414

[ref7] Garrido-Maestu A.; Prado M. Naked-eye Detection Strategies Coupled with Isothermal Nucleic Acid Amplification Techniques for the Detection of Human Pathogens. Compr. Rev. Food Sci. Food Saf. 2022, 21 (2), 1913–1939. 10.1111/1541-4337.12902. 35122372

[ref8] Charoenpanich P.; Mungkung A.; Seeviset N. A PH Sensitive, Loop-Mediated Isothermal Amplification Assay for Detection of Salmonella in Food. Sci. Eng. Health Stud. 2020, 14, 160–168.

[ref9] Brown T. A.; Schaefer K. S.; Tsang A.; Yi H. A.; Grimm J. B.; Lemire A. L.; Jradi F. M.; Kim C.; McGowan K.; Ritola K.; Armstrong D. T.; Mostafa H. H.; Korff W.; Vale R. D.; Lavis L. D. Direct Detection of SARS-CoV-2 RNA Using High-Contrast PH-Sensitive Dyes. J. Biomol. Tech. 2021, 32 (3), 121–133. 10.7171/jbt.21-3203-007. 35027870 PMC8730524

[ref10] Zasada A. A.; Wiatrzyk A.; Czajka U.; Brodzik K.; Formińska K.; Mosiej E.; Prygiel M.; Krysztopa-Grzybowska K.; Wdowiak K. Application of Loop-Mediated Isothermal Amplification Combined with Colorimetric and Lateral Flow Dipstick Visualization as the Potential Point-of-Care Testing for Corynebacterium Diphtheriae. BMC Infect. Dis. 2020, 20 (1), 308 10.1186/s12879-020-05037-z. 32334517 PMC7183728

[ref11] Tanner N. A.; Zhang Y.; Evans T. C. Visual Detection of Isothermal Nucleic Acid Amplification Using PH-Sensitive Dyes. Biotechniques 2015, 58 (2), 59–68. 10.2144/000114253. 25652028

[ref12] Yin K.; Pandian V.; Kadimisetty K.; Ruiz C.; Cooper K.; You J.; Liu C. Synergistically Enhanced Colorimetric Molecular Detection Using Smart Cup: A Case for Instrument-Free HPV-Associated Cancer Screening. Theranostics 2019, 9 (9), 2637–2645. 10.7150/thno.32224. 31131058 PMC6525999

[ref13] Lee J.-E.; Mun H.; Kim S.-R.; Kim M.-G.; Chang J.-Y.; Shim W.-B. A Colorimetric Loop-Mediated Isothermal Amplification (LAMP) Assay Based on HRP-Mimicking Molecular Beacon for the Rapid Detection of Vibrio Parahaemolyticus. Biosens. Bioelectron. 2020, 151, 111968 10.1016/j.bios.2019.111968. 31999578

[ref14] Daniel M.-C.; Astruc D. Gold Nanoparticles: Assembly, Supramolecular Chemistry, Quantum-Size-Related Properties, and Applications toward Biology, Catalysis, and Nanotechnology. Chem. Rev. 2004, 104, 293 10.1021/cr030698. 14719978

[ref15] Jazayeri M. H.; Aghaie T.; Avan A.; Vatankhah A.; Ghaffari M. R. S. Colorimetric Detection Based on Gold Nano Particles (GNPs): An Easy, Fast, Inexpensive, Low-Cost and Short Time Method in Detection of Analytes (Protein, DNA, and Ion). Sens. Bio-Sens. Res. 2018, 20, 1–8. 10.1016/j.sbsr.2018.05.002.

[ref16] Teixeira A.; Paris J. L.; Roumani F.; Diéguez L.; Prado M.; Espiña B.; Abalde-Cela S.; Garrido-Maestu A.; Rodriguez-Lorenzo L. Multifuntional Gold Nanoparticles for the SERS Detection of Pathogens Combined with a LAMP-in-Microdroplets Approach. Materials 2020, 13 (8), 1934 10.3390/ma13081934. 32325992 PMC7215531

[ref17] Arunrut N.; Kampeera J.; Suebsing R.; Kiatpathomchai W. Rapid and Sensitive Detection of Shrimp Infectious Myonecrosis Virus Using a Reverse Transcription Loop-Mediated Isothermal Amplification and Visual Colorogenic Nanogold Hybridization Probe Assay. J. Virol. Methods 2013, 193 (2), 542–547. 10.1016/j.jviromet.2013.07.017. 23876366

[ref18] Seetang-Nun Y.; Jaroenram W.; Sriurairatana S.; Suebsing R.; Kiatpathomchai W. Visual Detection of White Spot Syndrome Virus Using DNA-Functionalized Gold Nanoparticles as Probes Combined with Loop-Mediated Isothermal Amplification. Mol. Cell. Probes 2013, 27 (2), 71–79. 10.1016/j.mcp.2012.11.005. 23211683

[ref19] Zhang Y.; Chen M.; Liu C.; Chen J.; Luo X.; Xue Y.; Liang Q.; Zhou L.; Tao Y.; Li M.; Wang D.; Zhou J.; Wang J. Sensitive and Rapid On-Site Detection of SARS-CoV-2 Using a Gold Nanoparticle-Based High-Throughput Platform Coupled with CRISPR/Cas12-Assisted RT-LAMP. Sens. Actuators, B 2021, 345, 130411 10.1016/j.snb.2021.130411. PMC8257267 34248284

[ref20] López-Valls M.; Escalona-Noguero C.; Rodríguez-Díaz C.; Pardo D.; Castellanos M.; Milán-Rois P.; Martínez-Garay C.; Coloma R.; Abreu M.; Cantón R.; Galán J. C.; Miranda R.; Somoza Á.; Sot B. CASCADE: Naked Eye-Detection of SARS-CoV-2 Using Cas13a and Gold Nanoparticles. Anal. Chim. Acta 2022, 1205, 339749 10.1016/j.aca.2022.339749. 35414398 PMC8939626

[ref21] Zhang W. S.; Pan J.; Li F.; Zhu M.; Xu M.; Zhu H.; Yu Y.; Su G. Reverse Transcription Recombinase Polymerase Amplification Coupled with CRISPR-Cas12a for Facile and Highly Sensitive Colorimetric SARS-CoV-2 Detection. Anal. Chem. 2021, 93 (8), 4126–4133. 10.1021/acs.analchem.1c00013. 33570401

[ref22] Wong J. K. F.; Yip S. P.; Lee T. M. H. Ultrasensitive and Closed-Tube Colorimetric Loop-Mediated Isothermal Amplification Assay Using Carboxyl-Modified Gold Nanoparticles. Small 2014, 10 (8), 1495–1499. 10.1002/smll.201302348. 24623485

[ref23] Qin A.; Fu L. T.; Wong J. K. F.; Chau L. Y.; Yip S. P.; Lee T. M. H. Precipitation of PEG/Carboxyl-Modified Gold Nanoparticles with Magnesium Pyrophosphate: A New Platform for Real-Time Monitoring of Loop-Mediated Isothermal Amplification. ACS Appl. Mater. Interfaces 2017, 9 (12), 10472–10480. 10.1021/acsami.7b00046. 28276674

[ref24] Mori Y.; Hirano T.; Notomi T. Sequence Specific Visual Detection of LAMP Reactions by Addition of Cationic Polymers. BMC Biotechnol. 2006, 6 (1), 3 10.1186/1472-6750-6-3. 16401354 PMC1373654

[ref25] Tammam S. N.; Khalil M. A. F.; Abdul Gawad E.; Althani A.; Zaghloul H.; Azzazy H. M. E. Chitosan Gold Nanoparticles for Detection of Amplified Nucleic Acids Isolated from Sputum. Carbohydr. Polym. 2017, 164, 57–63. 10.1016/j.carbpol.2017.01.051. 28325344

[ref26] de Oliveira A. C.; Sabino R. M.; Souza P. R.; Muniz E. C.; Popat K. C.; Kipper M. J.; Zola R. S.; Martins A. F. Chitosan/Gellan Gum Ratio Content into Blends Modulates the Scaffolding Capacity of Hydrogels on Bone Mesenchymal Stem Cells. Mater. Sci. Eng., C 2020, 106, 110258 10.1016/j.msec.2019.110258. 31753363

[ref27] Bravo-Anaya L. M.; Soltero J. A.; Rinaudo M. DNA/Chitosan Electrostatic Complex. Int. J. Biol. Macromol. 2016, 88, 345–353. 10.1016/j.ijbiomac.2016.03.035. 27050113

[ref28] Bravo-Anaya L. M.; Fernández-Solís K. G.; Rosselgong J.; Nano-Rodríguez J. L. E.; Carvajal F.; Rinaudo M. Chitosan-DNA Polyelectrolyte Complex: Influence of Chitosan Characteristics and Mechanism of Complex Formation. Int. J. Biol. Macromol. 2019, 126, 1037–1049. 10.1016/j.ijbiomac.2019.01.008. 30615969

[ref29] Majdi H.; Salehi R.; Pourhassan-Moghaddam M.; Mahmoodi S.; Poursalehi Z.; Vasilescu S. Antibody Conjugated Green Synthesized Chitosan-Gold Nanoparticles for Optical Biosensing. Colloids Interface Sci. Commun. 2019, 33, 100207 10.1016/j.colcom.2019.100207.

[ref30] Yazid H.; Yassin A. M.; Ruslan A. Z.; Alias S. H.; Adnan R.; Md Jani A. M. Synthesis of Chitosan-Gold Nanoparticles for Drug Delivery. Adv. Mat. Res. 2014, 896, 280–283. 10.4028/www.scientific.net/AMR.896.280.

[ref31] Sun I.-C.; Na J. H.; Jeong S. Y.; Kim D.-E.; Kwon I. C.; Choi K.; Ahn C.-H.; Kim K. Biocompatible Glycol Chitosan-Coated Gold Nanoparticles for Tumor-Targeting CT Imaging. Pharm. Res. 2014, 31 (6), 1418–1425. 10.1007/s11095-013-1142-0. 23934255

[ref32] Tripathy S.; Chalana A. K.; Talukdar A.; Rajesh P. v.; Saha A.; Pramanik G.; Ghosh S. Limited-Resource Preparable Chitosan Magnetic Particles for Extracting Amplification-Ready Nucleic Acid from Complex Biofluids. Analyst 2022, 147 (1), 165–177. 10.1039/D1AN01150B. 34870658

[ref33] Gómez Pérez A.; González-Martínez E.; Díaz Águila C. R.; González-Martínez D. A.; González Ruiz G.; García Artalejo A.; Yee-Madeira H. Chitosan-Coated Magnetic Iron Oxide Nanoparticles for DNA and RhEGF Separation. Colloids Surf. A Physicochem. Eng. Asp. 2020, 591, 124500 10.1016/j.colsurfa.2020.124500.

[ref34] Pandit K. R.; Nanayakkara I. A.; Cao W.; Raghavan S. R.; White I. M. Capture and Direct Amplification of DNA on Chitosan Microparticles in a Single PCR-Optimal Solution. Anal. Chem. 2015, 87 (21), 11022–11029. 10.1021/acs.analchem.5b03006. 26439226

[ref35] Bakshi P. S.; Selvakumar D.; Kadirvelu K.; Kumar N. S. Chitosan as an Environment Friendly Biomaterial - a Review on Recent Modifications and Applications. Int. J. Biol. Macromol. 2020, 150, 1072–1083. 10.1016/j.ijbiomac.2019.10.113. 31739057

[ref36] da Silva A. B.; Rufato K. B.; de Oliveira A. C.; Souza P. R.; da Silva E. P.; Muniz E. C.; Vilsinski B. H.; Martins A. F. Composite Materials Based on Chitosan/Gold Nanoparticles: From Synthesis to Biomedical Applications. Int. J. Biol. Macromol. 2020, 161, 977–998. 10.1016/j.ijbiomac.2020.06.113. 32553969

[ref37] Diaz L.; Li Y.; Jenkins D. M. Chemical Stabilization of Dispersed Escherichia Coli for Enhanced Recovery with a Handheld Electroflotation System and Detection by Loop-Mediated Isothermal AMPlification. PLoS One 2021, 16 (1), e0244956 10.1371/journal.pone.0244956. 33400712 PMC7785231

[ref38] Lakshmi Narayanan R.; Sivakumar M. Preparation and Characterization of Gold Nanoparticles in Chitosan Suspension by One-Pot Chemical Reduction Method. Nano Hybrids 2014, 6, 47–57. 10.4028/www.scientific.net/NH.6.47.

[ref39] Papadakis G.; Pantazis A. K.; Fikas N.; Chatziioannidou S.; Tsiakalou V.; Michaelidou K.; Pogka V.; Megariti M.; Vardaki M.; Giarentis K.; Heaney J.; Nastouli E.; Karamitros T.; Mentis A.; Zafiropoulos A.; Sourvinos G.; Agelaki S.; Gizeli E. Portable Real-Time Colorimetric LAMP-Device for Rapid Quantitative Detection of Nucleic Acids in Crude Samples. Sci. Rep. 2022, 12 (1), 3775 10.1038/s41598-022-06632-7. 35260588 PMC8904468

[ref40] Zhang X.; Servos M. R.; Liu J. Ultrahigh Nanoparticle Stability against Salt, PH, and Solvent with Retained Surface Accessibility via Depletion Stabilization. J. Am. Chem. Soc. 2012, 134 (24), 9910–9913. 10.1021/ja303787e. 22646098

[ref41] Lang N. J.; Liu B.; Zhang X.; Liu J. Dissecting Colloidal Stabilization Factors in Crowded Polymer Solutions by Forming Self-Assembled Monolayers on Gold Nanoparticles. Langmuir 2013, 29 (20), 6018–6024. 10.1021/la3051093. 23617539

[ref42] Uribe-Alvarez C.; Lam Q.; Baldwin D. A.; Chernoff J. Low Saliva PH Can Yield False Positives Results in Simple RT-LAMP-Based SARS-CoV-2 Diagnostic Tests. PLoS One 2021, 16 (5), e0250202 10.1371/journal.pone.0250202. 33951060 PMC8099103

[ref43] Saxena A.; Pandey P.; Reddy N.; Rao V.; Chaudhary C. Estimation of Salivary Flow Rate, PH, Buffer Capacity, Calcium, Total Protein Content and Total Antioxidant Capacity in Relation to Dental Caries Severity, Age and Gender. Contemp. Clin. Dent. 2015, 6 (5), 65 10.4103/0976-237X.152943. PMC4374323 25821379

