# Supplementary material for: Naked-Eye Detection of LAMP-Produced Nucleic Acids in Saliva Using Chitosan-Capped AuNPs in a Single-Tube Assay
Source: Anal Chem. 2023 Dec 8;95(50):18514–21. doi: 10.1021/acs.analchem.3c03878 (PMC10733902; doi:10.1021/acs.analchem.3c03878)
Supplement: Supplementary file 1 — ac3c03878_si_001.pdf [file ac3c03878_si_001.pdf]

Supporting Information

Naked-Eye Detection of LAMP-Produced Nucleic Acids in Saliva using Chitosan-Capped AuNPs in a Single-Tube Assay

Stylianos Grammatikos [†,§], Ioannis Svoliantopoulos [‡,§], Electra Gizeli [*,†,§,]

[†] Department of Biology, University of Crete, 70013 Heraklion, Greece
[‡] Department of Chemistry, University of Crete, 70013 Heraklion, Greece
[§] Institute of Molecular Biology and Biotechnology, Foundation for Research and Technology-Hellas, 70013 Heraklion, Greece

Table of contents

Experimental section 3

1. LAMP primers sequences 3

2. Chit-AuNPs purification 3

3. Chit-AuNPs characterization 3

4. Synthesized Chit-AuNPs molar concentration calculation 4

Supporting Figures 5

S1. Schematic representation of the Chit-AuNPs synthesis reaction 5

S2. Spectroscopic characterization of the synthesized Chit-AuNPs 5

S3. Morphological characterization of the synthesized Chit-AuNPs 6

S4. LAMP reaction in the presence of Chit-AuNPs 6

S5. Purification of Chit-AuNPs via centrifugation 7

S6. Effect of time and pH on the stability of the final solutions 8

S7. Zeta-potential and UV-Vis absorbance of the final stable solutions 8

Supporting References 9

Experimental section

1. LAMP primers sequences

The set of six primers (100 µM) for the *Salmonella* invasion gene *invA* were purchased from Metabion (Germany), where their sequences (5'-3') were as follows:[1]

Inner FIP: *GACGACTGGTACTGATCGATAGTTTTTCAACGTTTCCTGCGG*
Inner BIP: *CCGGTGAAATTATCGCCACACAAAACCCACCGCCAGG*
Outer F3: *GGCGATATTGGTGTTTATGGGG*
Outer B3: *AACGATAAACTGGACCACGG*
Loop F: *GACGAAAGAGCGTGGTAATTAAC*
Loop B: *GGGCAATTCGTTATTGGCGATAG*

The set of six primers (100 µM) for the SARS-CoV-2 detection targeting the N gene were purchased from Eurofins Genomics (Germany), where their sequences were as follows:[2]

Inner FIP: *TGCGGCCAATGTTTGTAATCAGCCAAGGAAATTTTGGGGC*
Inner BIP: *CGCATTGGCATGGAAGTCACTTTGATGGCACCTGTGTAG*
Outer F3: *AACACAAGCTTTCGGCAG*
Outer B3: *GAAATTTGGATCTTTGTCATCC*
Loop F: *TTCCTTGTCTGATTAGTTC*
Loop B: *ACCTTCGGGAACGTGGTT*

2. Chit-AuNPs purification

Centrifugation was carried out using an Eppendorf 5417R Refrigerated Centrifuge. The 0.15% (w/v) Chit-AuNPs were centrifuged at appropriate conditions (volumes of 0.5 mL at 2000 rcf for 30 min, at 4 ^{o}C), in order to remove the free Chit from the solution. Afterward, redispersion of the pellet was performed in either (a) 1% (v/v) aqueous acetic acid; (b) ultrapure water; or (c) 1% (v/v) aqueous acetic acid containing 0.15% (w/v) Chit. The amount of excess Chit chosen for the redispersion is the same one used for the synthesis, so that it should naturally be higher than the free dissolved nonreacted Chit in the originally synthesized one. This purification step was carried out in order to study the effect of free Chit, as well as the pH of the final LAMP mixtures, after the addition of the Chit-AuNPs by spin-down.

3. Chit-AuNPs characterization

A variety of instrumental analytical methods were used to characterize the synthesized Chit-AuNPs. Initially, attenuated total reflectance/Fourier-transform infrared spectroscopy (ATR-FTIR) was used to semi-quantitatively measure the observable IR spectrum of the neat Chit and Chit-AuNPs by evaluating the transmittance over a spectral region of 4000 to 400 cm^{-1}. A VERTEX 70v FT-IR Spectrometer (Bruker), equipped with a A225/Q Platinum ATR unit with single reflection diamond crystal was used. To achieve a suitable signal quality, all spectra were collected at a resolution of 4 cm^{-1} by collecting 50 scans. Specimens were prepared by drop coating the studied solutions onto chemically cleaned glass substrates, and drying them at 60 ^{o}C for 30 min. The Chit-AuNPs formation was confirmed by observation of the surface plasmon resonance (SPR) band using UV-Visible absorption spectroscopy (Nanodrop ND-1000), by using 2 µL of each sample. The

Chit-AuNPs size and shape were studied by field emission gun - scanning electron microscopy (FEG-SEM, JSM-IT700HR, Jeol Ltd., Tokyo, Japan). Specimens for SEM measurements were prepared by drop coating diluted the colloidal solutions onto chemically cleaned silica substrates, and left to dry at ambient conditions. Image analysis of the SEM micrographs was performed using ImageJ software in order to calculate the average diameters of the synthesized Chit-AuNPs, where 100 NPs were measured from each sample. Finally, the z-average (intensity weighted mean hydrodynamic diameter (D_h)), polydispersity index (PDI) and mean z-potential values of the studied solutions were determined by dynamic light scattering (DLS) and zeta potential (ZP) measurements, using a Malvern Zetasizer Nano ZS90 (Malvern Instruments Ltd, UK) after diluting them with ultrapure water, while measurements were performed in triplicate.

4. Synthesized Chit-AuNPs molar concentration calculation

The mean core size of the three Chit-AuNPs solutions was calculated by analyzing the SEM micrographs using the ImageJ software, as previously mentioned. The mean Chit-AuNPs diameter (D) calculated were: 38.4, 13.8 and 10.7 nm for Chit-AuNPs synthesized using 0.15%, 0.25% and 0.35% (w/v) Chit, respectively. For the Chit-AuNPs concentration calculation, three assumptions are made: i) $HAuCl_4$ is entirely converted into AuNPs, ii) density (ρ) of AuNPs is equal to density of bulk Au (19.3 g/cm^3) and iii) AuNPs are spherical in shape with a uniform FCC crystal structure.[3]
The average number of gold atoms (N) per nanoparticle can be expressed as:

$$N = \frac{\pi \rho N_A D^3}{6M}$$

where M= 197 g/mol the molar mass of Au and N_A: Avogadro's number, while the concentration (C) of the nanoparticles inside the solution as:

$$C = \frac{moles\ from\ reaction}{NV}$$

where V: total reaction volume (in liters).
The molar concentrations (C) were calculated as: 0.57, 12.3 and 26.4 nM for Chit-AuNPs synthesized using 0.15%, 0.25% and 0.35% (w/v) Chit, respectively.

Supporting Figures

S1. Schematic representation of the Chit-AuNPs synthesis reaction

The Chit-AuNPs "green" synthesis employed in this study follows a chemical reduction process, where chitosan has a double role of a reducing and capping/stabilizing agent. Upon protonation of their amine groups in the presence of acetic acid, chitosan chains electrostatically adsorb negatively charged Au (III) ions that are reduced to neutral Au atoms, and further agglomerate to form AuNPs capped with chitosan.[4]

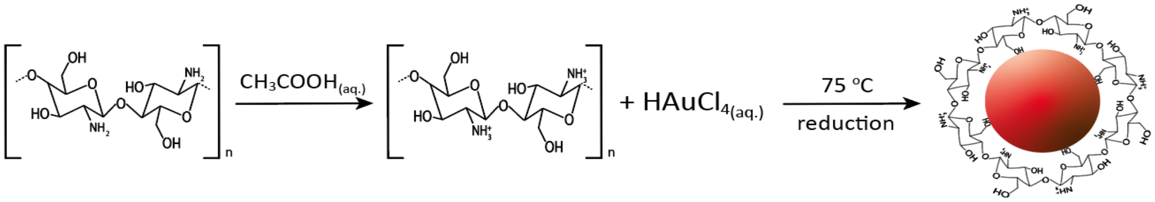

Figure S1: Schematic representation of AuNPs formulation using Chit dissolved in 1% (v/v) aqueous acetic acid.

S2. Spectroscopic characterization of the synthesized Chit-AuNPs

ATR-FTIR spectroscopy was conducted for neat Chit and Chit-AuNPs samples, in order to study the interaction between the Chit polymer and the formed AuNPs. The characteristic absorption bands from the functional groups of neat Chit observed (S2a) were identified based on the literature.[5] When comparing the neat Chit graph with the Chit-AuNPs one, a decrease in the intensity of several peaks, as well as shifts to lower energies were observed in the latter case. Based on these results, a contact between Chit and AuNPs through different chemical groups can be concluded (supramolecular interaction), in agreement with previous reports.[6,7] As shown in the UV-Vis absorption spectra of the synthesized Chit-AuNPs (S2b), all the solutions have SPR bands, with the SPR peaks (λ_{max}) at 532, 523 and 520 nm for 0.15%, 0.25% and 0.35% (w/v) Chit-AuNPs, respectively. This implies an inversely proportional relation between the Chit concentration and the synthesized Chit-AuNPs sizes, in agreement with similar studies.[8,9] Regarding the full width at half maximum (FWHM), it is larger in the lowest Chit concentration, implying bigger polydispersity in the Chit-AuNPs sizes.[10] DLS and ZP measurements revealed a mean D_h of 59.6, 50.4 and 64.1 nm, PDI values at 0.46, 0.42 and 0.29, and mean z-potential values of +45.7, +46.4 and +53.4 mV for the Chit-AuNPs synthesized using 0.15%, 0.25% and 0.35% (w/v) Chit, respectively (S2b). In general, the calculated D_h is higher than the NPs size calculated via microscopy techniques, owing to the hydration shell around the NPs affecting their diffusion speed, and by extension their calculated size.[11] Regarding the PDI, it is higher in the Chit-AuNPs solution synthesized with the lowest Chit concentration (0.15%, w/v), in agreement with the above-mentioned conclusions based on the FWHM of the UV-Vis spectra. Z-potential values are positive owing to the cationic nature of Chit in the acidic solution, with values greater than 25 mV, implying a high degree of stability for all the colloidal solutions. Notably, the above-mentioned values are also affected by the presence of free dissolved Chit in the solutions, that may undergo self-crosslinking to produce Chit polymer chains.[11]

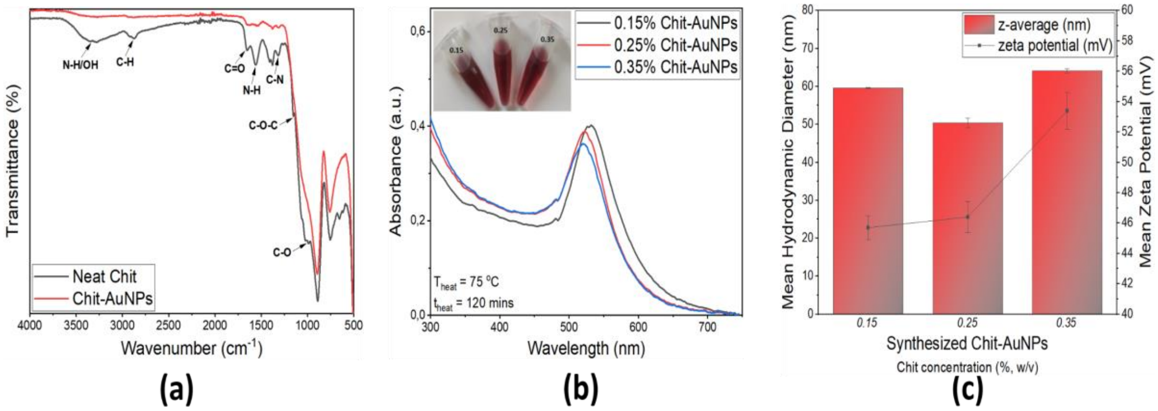

Figure S2. a) Representative ATR-FTIR spectra of neat Chit (black line) and Chit-AuNPs (red line), along with the peaks of interest. b) UV-Vis absorbance spectra and c) DLS and ZP measurements of the Chit-AuNPs synthesized with different Chit concentrations (0.15%, 0.25% and 0.35%, w/v) at 75 ^{o}C for 2h.

S3. Morphological characterization of the synthesized Chit-AuNPs

SEM images were used to study the morphology of the synthesized Chit-AuNPs. It can be seen that the Chit-AuNPs synthesized with the lowest Chit concentration comprise larger and more polydisperse NPs, in agreement with the results of the spectroscopic analysis of the colloidal solutions. Besides spherical ones, thin triangular prismatic AuNPs can be observed, particularly in the Chit-AuNPs synthesized with the lowest Chit concentration, possibly due to the slower AuNPs growth process.[12]

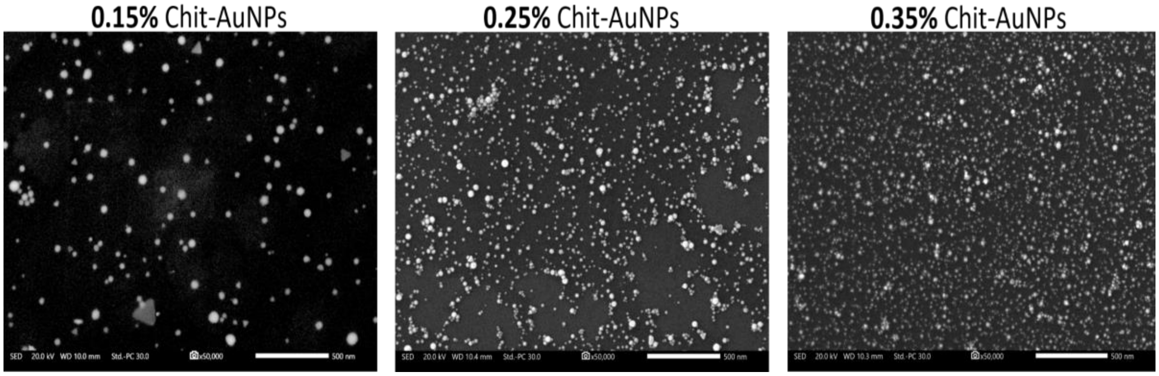

Figure S3. SEM images of the Chit-AuNPs synthesized with different Chit concentrations (0.15%, 0.25% and 0.35%, w/v) at 75 ^{o}C for 2h (scale bar: 500 nm).

S4. LAMP reaction in the presence of Chit-AuNPs

Addition of neat Chit or Chit-AuNPs directly inside the LAMP mix completely inhibits the amplification reaction (S4a), owing to their electrostatic interactions with LAMP reagents (final solution pH~4.5). In these reactions, 5 µL of nuclease-free water were replaced by either 0.15% (w/v) neat Chit or Chit-AuNPs, and 0.5 µL of nuclease-free water by 0.5 µL of LAMP fluorescent dye. Picture (S4b) shows the tubes after the LAMP reaction. In the normal fluorescence assay, the negative sample remains transparent while the positive (100 cfu/reaction *Salmonella*) is blurrier owing to the increase of turbidity because of LAMP's $Mg_2P_2O_7$ byproduct.[13] When incorporating neat Chit inside the solutions, both negative and positive samples appear blurry due to the presence of free Chit. In the Chit-AuNPs case, a visible pellet is formed possibly due to the Chit-AuNPs heat-induced aggregation inside the LAMP mix, as Chit-AuNPs obtain enough energy (63 ^{o}C heating) to cross the free Chit's depletion barrier.[14]

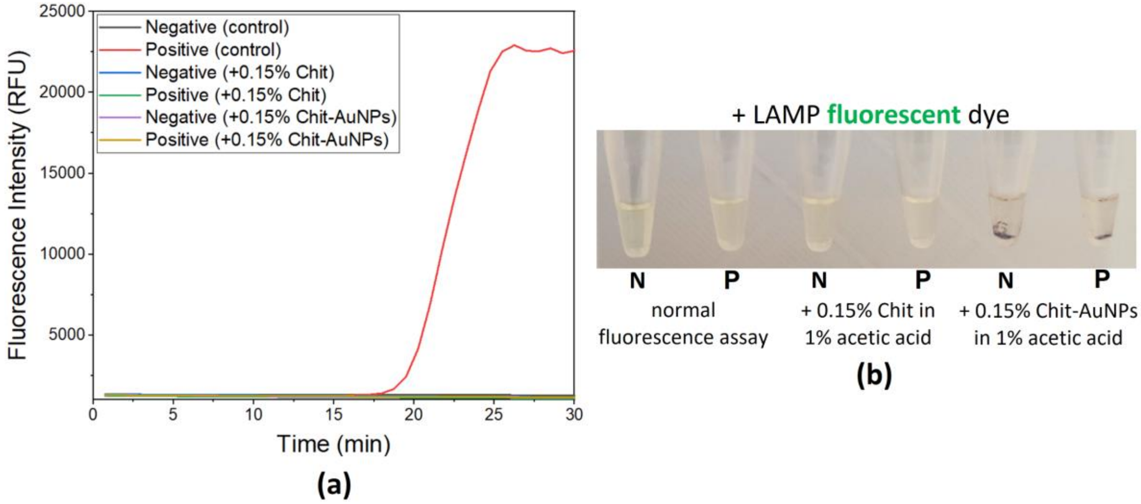

Figure S4. a) Real time fluorescence LAMP using the fluorescent dye. Addition of neat Chit or Chit-AuNPs completely inhibits the LAMP reaction. b) Photograph of the tubes after LAMP reaction. No apparent colorimetric difference can be observed between the negative and positive samples when neat Chit or Chit-AuNPs are included, due to the inhibition of the amplification reaction. Note: the N down the photograph of the 0.2 mL tubes corresponds to negative samples, while the P corresponds to positives with 100 cfu/reaction (*Salmonella*).

S5. Purification of Chit-AuNPs via centrifugation

The Chit-AuNPs synthesized using 0.15% (w/v) Chit were centrifuged, in order to purify the solution by removing the free dissolved nonreacted Chit. After redispersion in the different chosen media, no color change was observed, indicating no aggregation during the purification step. This is also confirmed via the UV-Vis spectra (S5a), where no red shifting in the SPR λ_{max} was observed. Furthermore, measurements of the D_h and z-potential (S5b) of the resulting solutions revealed a significant increase in the case of excess Chit addition in both parameters (159.7 nm mean D_h and +68.4 mV z-potential) compared to the originally synthesized solution (59.6 nm mean D_h and +45.7 mV z-potential), possibly reflecting free Chit's dominating presence inside the solution. The solutions without free Chit (redispersion in 1% (v/v) aqueous acetic acid or ultrapure water) didn't present significant differences in the z-potential values when compared to the originally synthesized solution, while the D_h showed a decrease of about 16 nm in both cases.

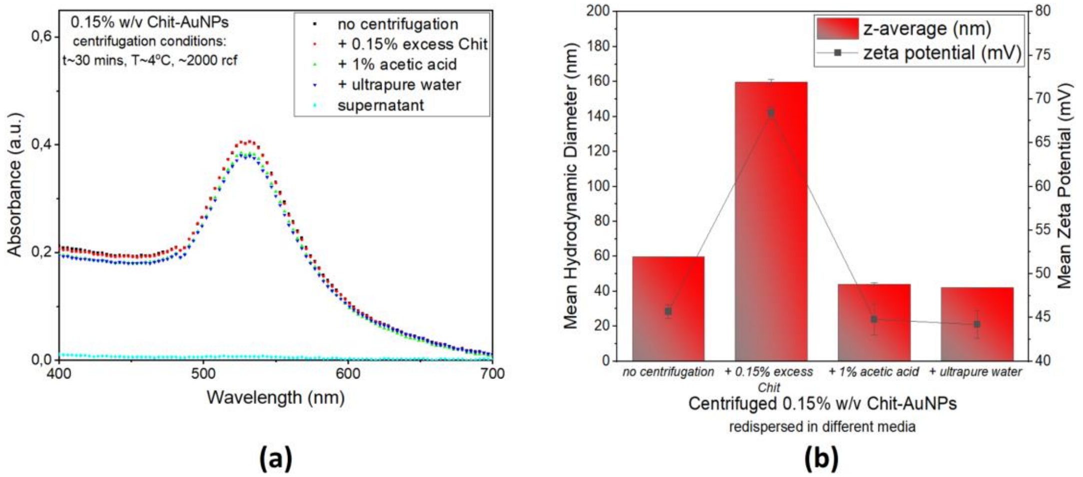

Figure S5. a) UV-Vis spectra, b) DLS and ZP measurements, of the 0.15% (w/v) Chit-AuNPs, after centrifugation and redispersion in different solvents (0.15% (w/v) Chit in 1% (v/v) aqueous acetic acid, 1% (v/v) aqueous acetic acid, ultrapure water).

S6. Effect of time and pH on the stability of the final solutions

An ageing test in assays using 20% saliva revealed that when using free Chit, the negatives also tend to aggregate after some time (~4 days), possibly due to the differently charged populations inside the solution. At the same time, the aggregation in the positive samples happened significantly faster (immediately after the spin-down/mixing of the Chit-AuNPs and LAMP solutions), therefore there was no problem differentiating the positive samples from the negative ones. In this assay, the pellet formed is initially a Chit-DNA electrostatic complex,[15] which subsequently is further enriched by Chit-AuNPs, and hence the pellet appearance inside the tube. In the assays without free Chit, the positive samples remain stable over time, owing to DNA-induced electrosteric stabilization of Chit-AuNPs inside the LAMP mix.[14] In this assay, the pellet formed in the negative samples is due to Chit-AuNPs aggregation induced by the different LAMP reagents, as there is not enough DNA present in the solution to efficiently stabilize them by electrostatic conjugation (S6a). In the case of removal of free Chit and redispersion of 0.15% (w/v) Chit-AuNPs in ultrapure water instead of 1% (v/v) aqueous acetic acid, aggregation in both negative and positive samples was observed. This indicates that when the pH of the final solution (Chit-AuNPs + LAMP) is close to or higher than Chit's pKa of ~6.5,[16] the electrostatic interactions between the Chit-AuNPs and the amplified DNA may not be favored owing to the partial neutralization of Chit-AuNPs charge. This reduction of electrostatic stabilization (or repulsive Coulomb forces) between the Chit-AuNPs essentially leaves only the steric stabilization provided by the Chit coating of the AuNPs present in the solution, so they also tend to aggregate in the positive samples owing to the dominating attractive Van der Waals forces inside the LAMP mix (S6b), in the absence of strong Chit-DNA electrostatic interactions.

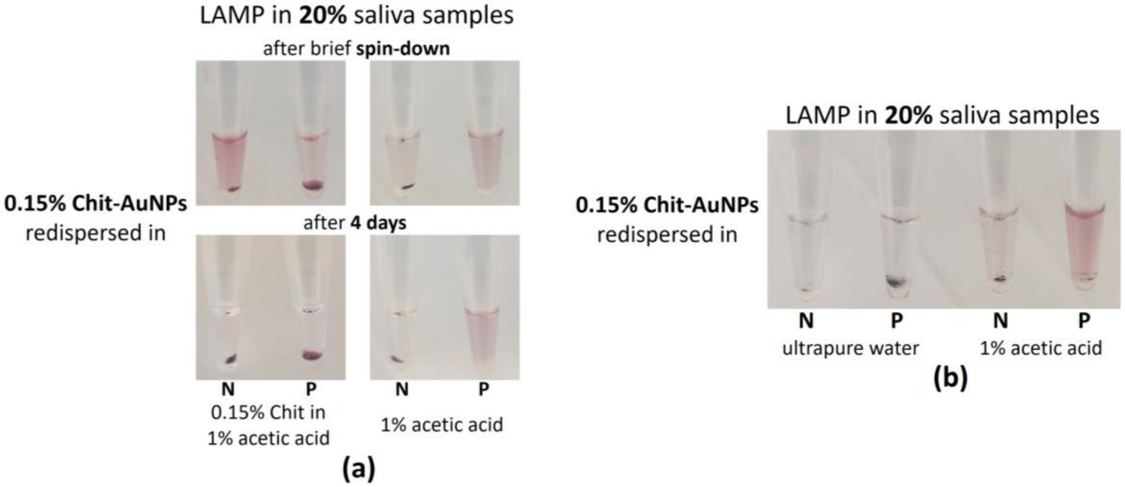

Figure S6. a) Effect of time on the stability of LAMP reaction-Chit-AuNPs mix after amplification, in the presence of 20% saliva samples. In the assay using free Chit, the stable negative is gradually destabilized over time, while in the assay without free Chit, the stable positive maintains its stability over time. b) Effect of pH on the redispersion medium of centrifuged 0.15% (w/v) Chit-AuNPs. An acidic pH is preferable instead of a neural for a visible colorimetric difference between negative and positive samples, in order for electrostatic interactions between Chit-AuNPs and DNA to occur. Note: the N down the photographs of the 0.2 mL tubes corresponds to negative samples, while the P corresponds to positives with 100 cfu/reaction (*Salmonella*).

S7. Zeta-potential and UV-Vis absorbance of the final stable solutions

In the assay using Chit-AuNPs with free Chit, the stable negative sample presents a positive z-potential value (~ +10.7 mV), meaning that the supernatant's stability is due to the dominating

presence of free Chit in the solution which hasn't been completely saturated. In the assay using Chit-AuNPs only, the stable positive sample presents a negative z-potential value (~ -7.1 mV), indicative of the efficient electrostatic conjugation of positively charged Chit-AuNPs with negatively charged amplified DNA (S7a). This conjugation is further confirmed by the observed red-shifting in the UV-Vis spectra, where the stable positive solution presented a redshift of the SPR λ_{max} to 541 nm and a drop in the absorbance intensity, compared to the stable negative sample that presented a λ_{max} of 532 nm (S7b).

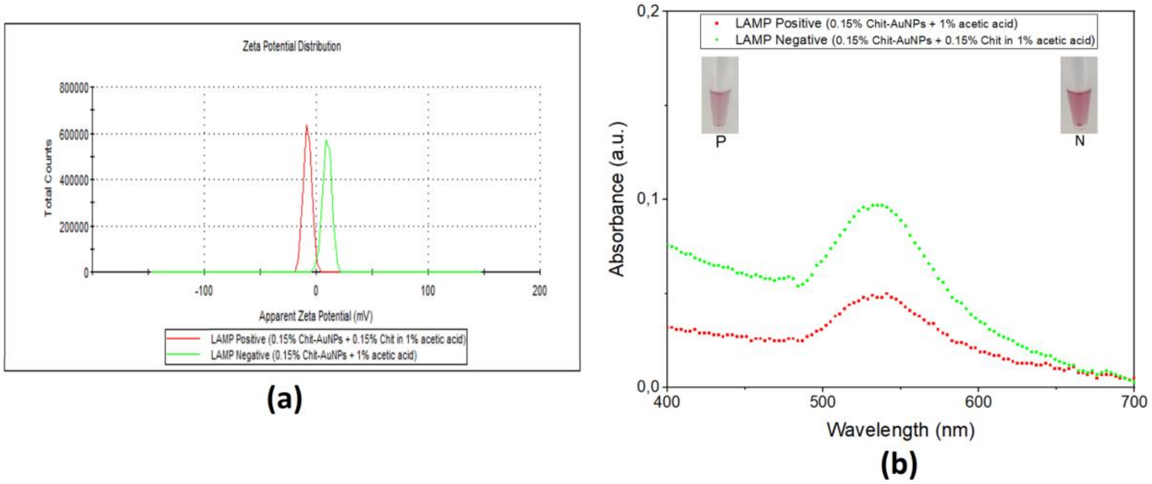

Figure S7. a) Zeta potential distribution and b) UV-Vis absorbance spectra, of a stable negative (free Chit/Chit-AuNPs + LAMP) and a stable positive (Chit-AuNPs + LAMP) sample. The stable negative presented a positive z-potential (~ +10.7 mV) and an SPR λ_{max} at 532 nm, while the stable positive presented a negative z-potential (~ -7.1 mV) and an SPR λ_{max} at 541 nm, indicative of the efficient conjugation of the amplified DNA to the Chit-AuNPs surface in the latter case.

Supporting References

(1) Hara-Kudo, Y.; Yoshino, M.; Kojima, T.; Ikedo, M. Loop-Mediated Isothermal Amplification for the Rapid Detection of Salmonella. *FEMS Microbiol Lett* **2005**, *253* (1), 155–161. https://doi.org/10.1016/j.femsle.2005.09.032.

(2) Broughton, J. P.; Deng, X.; Yu, G.; Fasching, C. L.; Servellita, V.; Singh, J.; Miao, X.; Streithorst, J. A.; Granados, A.; Sotomayor-Gonzalez, A.; Zorn, K.; Gopez, A.; Hsu, E.; Gu, W.; Miller, S.; Pan, C.-Y.; Guevara, H.; Wadford, D. A.; Chen, J. S.; Chiu, C. Y. CRISPR–Cas12-Based Detection of SARS-CoV-2. *Nat Biotechnol* **2020**, *38* (7), 870–874. https://doi.org/10.1038/s41587-020-0513-4.

(3) Khashayar, P.; Amoabediny, G.; Larijani, B.; Hosseini, M.; Vanfleteren, J. Fabrication and Verification of Conjugated AuNP-Antibody Nanoprobe for Sensitivity Improvement in Electrochemical Biosensors. *Sci Rep* **2017**, *7* (1), 16070. https://doi.org/10.1038/s41598-017-12677-w.

(4) Lakshmi Narayanan, R.; Sivakumar, M. Preparation and Characterization of Gold Nanoparticles in Chitosan Suspension by One-Pot Chemical Reduction Method. *Nano Hybrids* **2014**, *6*, 47–57. https://doi.org/10.4028/www.scientific.net/NH.6.47.

(5) Fernandes Queiroz, M.; Melo, K.; Sabry, D.; Sassaki, G.; Rocha, H. Does the Use of Chitosan Contribute to Oxalate Kidney Stone Formation? *Mar Drugs* **2014**, *13* (1), 141–158. https://doi.org/10.3390/md13010141.

(6) Silva, A. T. B.; Coelho, A. G.; Lopes, L. C. da S.; Martins, M. V. A.; Crespilho, F. N.; Merkoçi, A.; Silva, W. C. da. Nano-Assembled Supramolecular Films from Chitosan-Stabilized Gold Nanoparticles and Cobalt(II) Phthalocyanine. *J Braz Chem Soc* **2013**, *24* (8), 1237–1245. https://doi.org/10.5935/0103-5053.20130157.

(7) Thanayutsiri, T.; Patrojanasophon, P.; Opanasopit, P.; Ngawhirunpat, T.; Plianwong, S.; Rojanarata, T. Rapid Synthesis of Chitosan-Capped Gold Nanoparticles for Analytical Application and Facile Recovery of Gold from Laboratory Waste. *Carbohydr Polym* **2020**, *250*, 116983. https://doi.org/10.1016/j.carbpol.2020.116983.

(8) Huang, H.; Yang, X. Synthesis of Chitosan-Stabilized Gold Nanoparticles in the Absence/Presence of Tripolyphosphate. *Biomacromolecules* **2004**, *5* (6), 2340–2346. https://doi.org/10.1021/bm0497116.

(9) Sun, L.; Li, J.; Cai, J.; Zhong, L.; Ren, G.; Ma, Q. One Pot Synthesis of Gold Nanoparticles Using Chitosan with Varying Degree of Deacetylation and Molecular Weight. *Carbohydr Polym* **2017**, *178*, 105–114. https://doi.org/10.1016/j.carbpol.2017.09.032.

(10) Oliveira, J. P.; Prado, A. R.; Keijok, W. J.; Ribeiro, M. R. N.; Pontes, M. J.; Nogueira, B. V.; Guimarães, M. C. C. A Helpful Method for Controlled Synthesis of Monodisperse Gold Nanoparticles through Response Surface Modeling. *Arabian Journal of Chemistry* **2020**, *13* (1), 216–226. https://doi.org/10.1016/j.arabjc.2017.04.003.

(11) Komalam, A.; Muraleegharan, L. G.; Subburaj, S.; Suseela, S.; Babu, A.; George, S. Designed Plasmonic Nanocatalysts for the Reduction of Eosin Y: Absorption and Fluorescence Study. *Int Nano Lett* **2012**, *2* (1), 26. https://doi.org/10.1186/2228-5326-2-26.

(12) Potara, M.; Maniu, D.; Astilean, S. The Synthesis of Biocompatible and SERS-Active Gold Nanoparticles Using Chitosan. *Nanotechnology* **2009**, *20* (31), 315602. https://doi.org/10.1088/0957-4484/20/31/315602.

(13) Mori, Y.; Kitao, M.; Tomita, N.; Notomi, T. Real-Time Turbidimetry of LAMP Reaction for Quantifying Template DNA. *J Biochem Biophys Methods* **2004**, *59* (2), 145–157. https://doi.org/10.1016/j.jbbm.2003.12.005.

(14) Shin, J.; Zhang, X.; Liu, J. DNA-Functionalized Gold Nanoparticles in Macromolecularly Crowded Polymer Solutions. *J Phys Chem B* **2012**, *116* (45), 13396–13402. https://doi.org/10.1021/jp310662m.

(15) Amaduzzi, F.; Bomboi, F.; Bonincontro, A.; Bordi, F.; Casciardi, S.; Chronopoulou, L.; Diociaiuti, M.; Mura, F.; Palocci, C.; Sennato, S. Chitosan-DNA Complexes: Charge Inversion and DNA Condensation. *Colloids Surf B Biointerfaces* **2014**, *114*, 1–10. https://doi.org/10.1016/j.colsurfb.2013.09.029.

(16) de Oliveira, A. C.; Sabino, R. M.; Souza, P. R.; Muniz, E. C.; Popat, K. C.; Kipper, M. J.; Zola, R. S.; Martins, A. F. Chitosan/Gellan Gum Ratio Content into Blends Modulates the Scaffolding Capacity of Hydrogels on Bone Mesenchymal Stem Cells. *Materials Science and Engineering: C* **2020**, *106*, 110258. https://doi.org/10.1016/j.msec.2019.110258.
